# Grade Retention in Primary Education Is Associated with Quarter of Birth and Socioeconomic Status

**DOI:** 10.1371/journal.pone.0166431

**Published:** 2016-11-16

**Authors:** Sara M. González-Betancor, Alexis J. López-Puig

**Affiliations:** 1 Economía de la Salud y Políticas Públicas, Departamento de Métodos Cuantitativos en Economía y Gestión, Universidad de Las Palmas de Gran Canaria, ULPGC, Las Palmas de Gran Canaria, Spain; 2 Departamento de Ingeniería Electrónica y Automática, Universidad de Las Palmas de Gran Canaria, ULPGC, Las Palmas de Gran Canaria, Spain; 3 Agencia Canaria de Calidad Universitaria y Evaluación Educativa (ACCUEE), Gobierno de Canarias, Spain; University of Westminster, UNITED KINGDOM

## Abstract

Grade retention is still common practice in some countries though longstanding experience tells us that it is a highly criticised practice for its unclear benefits, its important costs for the educational systems and its relation with school dropout. Therefore, the aim of the present study is to analyse which variables increase the probability of being retained in primary education differentiating between being retained in second or in fourth grade, and paying special attention to the role of the socioeconomic status of the families. By knowing which analysed variables are related to grade retention, and how, we may offer some suggestions to reduce it. We use a national dataset with more observations for Spain than any other international ones, called ‘*Evaluación General de Diagnóstico*’, conducted in Spain in 2009 with the participation of 28708 students of fourth grade of primary education from 874 schools, considered to be representative for every Spanish autonomous region. This assessment focused on four competences and includes information about the learning context collected through questionnaires for students, families, school management and teachers. Estimating different multilevel random-intercept logistic regressions we obtain the following three main findings: 1) the existence of a ‘quarter of birth’ effect, that nearly doubles the probability of grade retention in second grade of primary –compared to the probability of grade retention in fourth grade–, for the youngest students of their same age cohort (OR = 1.93 vs. OR = 1.53, both p<0.001); 2) that the mothers’ education level influences more than the fathers’ one –especially in second grade (OR = 0.20 vs. OR = 0.45, both p<0.001)–; and 3) that having an unemployed father increases the probability of grade retention much more than having an unemployed mother –especially in second grade (OR = 1.48, p<0.005 vs. OR = 1.18, p>0.05)–.

## Introduction

Grade retention, i.e. the practice of having students repeat a year of schooling in which they did not meet certain educational or social standards, is a common practice in some countries –e.g. Portugal, Spain, France and Belgium–, an exceptional practice in others –e.g. Finland, Poland, Greece– and in others grade retention is not an option –e.g. Norway, Island, United Kingdom–[1]. Though grade retention is a longstanding experience in some countries, it is a practice that has been highly criticised for its unclear benefits and its wellknown drawbacks.

Regarding the possible effects of grade retention in learning improvement, as well as in the subsequent educational performance and in other social and emotional aspects, a meta-analysis of 22 studies [2] shows the importance of the design quality of the research carried out, and the necessity of including the time passed since retention, as both aspects are associated with a less negative effect of grade retention. Goos, Van Damme, Onghena, Petry, & De-Bilde [3], point out that the effect of grade retention is less useful than expected by parents and educators, and Goos, Belfi, et al. [4] obtain the same result focusing on the long term. In fact, other studies point to the need of analysing the effects of grade retention differentiating between the short, medium and long run. Regarding the short and medium run, Bonvin et al. [5] find a positive effect of grade retention when comparing students of the same grade, while it is negative when this comparison is made in terms of students of the same age. Others [6, 7] find that it is not a lasting effect, or that it does have a lasting effect merely in the social acceptance of the student [8]. Furthermore, a positive effect of grade retention could be related to an improvement in attention problems, but not in academic results [9].

Regarding the negative effect of grade retention in the subsequent educational performance, there is evidence that it extends to the fifth grade –if repetition took place in preschool– or during three courses –if repetition occurred in the first grade of primary education–[10], i.e. until nearly the end of the stage in both cases. Other works support these findings, like [11], where the negative effect of retention in second grade of primary lasts at least until fourth grade. In the case of repetition during secondary education it can also last from four to five years [10].

Other effects of grade retention are related to an increase in the likelihood of dropout in secondary education [12], whether the grade repetition took place in previous stages –i.e. in preschool or primary education [13, 14]–or it took place during secondary education [15]. Especially when it takes place during primary education, other related effects, like anxiety and disruptive behaviors, persist later on [16]. However, it does not seem to have a negative effect on the social and emotional development [17], or on the motivation of students [18], but on parental expectations [19].

Despite not having met the previous grade standards, students’ promotion to the next grade with their same-age peers is the alternative to grade retention. Looking for the consequences of this alternative, the findings are not conclusive. Depending on the data used, some studies indicate that students would have learned more, had they been promoted [20], or even, that repeaters are more likely to get better results in external assessments, as if they had been promoted [21].

Although some authors have concluded recently that there is no negative effect of grade retention, and that the incompliance of this result with the previous literature may be due to methodological issues [22], there is no doubt that it is not satisfactory, so researchers, educators and legislators should look for other more productive alternatives instead of repetition [23]. In the same vein Reschly & Christenson [24] indicate that the real issue is to analyse what strategies should be followed with students who do not meet certain educational or social standards, advancing other complementary proposals like giving carefully monitored instructions and supplementary interventions that address the student’s learning needs.

The decision to retain a student is subjective, usually conducted by a team of teachers, and often not even with the support of the school psychologists [25]. Even the belief that retention in the early primary grades may be exceptionally positive, while the one at the end of this stage is not, is a non-solid argument as well [26]. When retained, students in kindergarten do not get academic benefits of this grade retention. In fact, they will be able to improve their performance, if they are promoted to primary education with educational support measures [27]. Some studies point to the fact that grade retention at such a young age could be directly related to the need of specific educational support that has not yet been considered [28, 29] or that it could be associated with health problems [30]. Furthermore, the effect of grade retention differs wether it takes place in primary or in secondary education, having a better effect in the latter case [31], at least in Spain.

The decision to repeat is generally related to the student characteristics, such as low academic performance [32, 33], even at the beginning of primary education [34], and is usually related with gender and areas like reading and mathematics [35, 36]. It is also related with immigrant conditions. In fact, when grade retention is analysed from the point of view of immigration, evidence shows that first-generation immigrant students are at greater risk of repeating than natives, while second-generation immigrant students with similar characteristics are less likely to repeat [37].

But grade retention has been proved to be driven by another type of characteristics, such as the teachers’ attitude and evaluation [5], the low sense of responsibility of parents regarding their children’s school education [38], having older classmates [39], belonging to a low income family [40], the constraints and preferences of local constituencies and leadership [41] or the traditions and beliefs of each country [42].

Another variable, called ‘season of birth effect’ or ‘quarter of birth effect’ [11, 43], referring to the fact that the youngest children within a class are disadvantaged when compared to their older classmates, has also been found to influence childrens’ success in schools, and consequently implies grade retention [20, 34]. Likewise, family socioeconomic status influences students’ success, measured in terms of the scores obtained in international evaluations [12] and grade retention [44]. Furthermore, not only does family socioeconomic status influence students’ success, but also the socioeconomic status of the educational centre itself. As a matter of fact, it has been found out that the students of schools with a higher proportion of less-advantageous socioeconomic status show worst academic results [44].

In Spain students may be retained in primary education by law only once, if they do not show to have the required basic competences of the specific educational level, or if they do not achieve the objectives in more than two academic fields. Grade retention is not only possible in primary education in Spain, but it is also a used practice, though there is no clear evidence of its benefits. The phenomenon of grade retention in primary education is a reason of concern in Spain, as it affects 4.7% (4.0% and 4.5%) of the students in second (fourth and sixth) grade. Therefore, the suitable rate in primary education in Spain, that is, the percentage of students enrolled in its corresponding grade according to their age, is around 94% (89% and 84%) for the students with 8 (10 and 12) years old [45]. The decision of retaining a student in second grade leaves no possibility of being retained in any other grade during primary education. Therefore, though this student may have future difficulties in the learning process, he/she will be promoted automatically, until he/she finishes primary education. This may lead to grade retention during secondary education, as the student may have a learning gap that makes it difficult for him/her to achieve the academic competences of secondary education.

Just from an economic point of view, grade retention has far-reaching economic and social implications, because being one extra year in the education system means, at least, one more year of educational costs, as well as entering the labour market at least one year later, and probably obtaining lower paid jobs. Grade retention is also related to school dropout, which could mean entering the labor market without the required qualifications. Therefore, it becomes important to every Economy to analyse which variables increase the probability of being retained. Knowing which features influence grade retention, and how they do so, there is a possibility of detecting which ones might be addressed by policy makers, or even by teachers or families.

In Spain, there are previous studies that deal with this issue by analysing the determinants of grade retention with PISA data [46–49] or even with PIRLS data [50]. Others focus their interest in the relation between grade retention and school failure using as well different waves of PISA data [51, 52]. But all of them show the same limitation derived from the datasets they use. Those who used the first waves of PISA had to use a proxy for retention by checking for age mismatch. Since 2003 there has been a direct question that asks students if they were retained in primary or secondary education. But, until the last wave, there has been no variable in these datasets –neither in PISA, nor in PIRLS or even TIMMS– with the information about the exact grade the students were retained.

The aim of the present study is to go a little bit further than other previous studies, and analyse, for the first time –at least for Spain–, which variables increase the probability of being retained in primary education, differentiating between being retained in second or in fourth grade, and paying special attention to the role of socioeconomic status (hereafter SES).

Previous studies have shown that socioeconomic status explains academic results [53, 54] –and consequently grade retention– to a great extent [55–58]. But this status is usually measured with a previously built index that sums up a lot of different family characteristics. In the present work, apart from using an index, we deepen on it and analyse which of those characteristics influence the probability of being retained.

## Material and Methods

### The sample

The Spanish education law of 2006 established the compulsory diagnostic assessment of fourth grade students of primary education. This measure aimed at 1) improving educational standards and equity, 2) guiding educational policies, 3) increasing transparency and the efficiency of the education system and 4) offering information about the degree of acquisition of the key competences. This assessment would either be sample-based to obtain representative results of the students and the schools of each autonomous region –called ‘*Evaluación General de Diagnóstico* (EGD)’–or census-based, thus conducting it among all fourth grade students of primary education –called ‘*Evaluación de Diagnóstico* (ED)’–.

The EGD2009 focused on the communicative linguistic competence (CLC), the mathematic competence (MC), the competence in knowledge and interaction with the physical world (CKIPW) and the competence in social skills and citizenship (CSSC) of fourth grade students of primary education. To assess the level of development in these key competences the students had 50 minutes to answer questions in a booklet with different degrees of difficulty. The tests were corrected on the basis of the Item Response Theory, so that an outcome per student and competence was obtained. The results of the tests, as in PISA (Programme for International Student Assessment), were standardized with a global mean of 500 points and a standard deviation of 100.

The sample was obtained from a two-stage fixed stratified cluster sample design, considering the autonomous regions as the strata. The cluster comprises the schools and, if so, the students in the same classroom. The first stage is the random selection of schools (about 50 in every autonomous region), conditioned by their size and ensuring that the sample obtained is representative for the whole of Spain and for each autonomous region. During the second stage there is a random selection of one or two groups of fourth grade of primary among schools with more than one group of students per year.

The sample obtained in the 2009 tests covers 28708 students from 874 schools, assuming a maximum sampling error of 3% [59]. The EGD2009 also includes information about the learning context collected through questionnaires for students, families, school management (874 heads of school) and teachers (1341). This national dataset has more observations for Spain than any other international one, thus favouring more robust analysis and results. In the case of PISA, the Spanish students’ sample was 10791, 19604, 25887 and 25312 in 2003, 2006, 2009 and 2012, respectively. PIRLS has smaller samples (4360 in 2006 and 8580 in 2011) and TIMSS, even smaller (4183 in 2011). There is also another important difference compared to other international datasets, that is, the sample is considered to be representative for every Spanish autonomous region.

Focusing on grade retention, the EGD2009 shows that 92.3% of the students has never been retained until fourth grade of primary, whereas 7.7% already had. In fact, 3.7% were retained in second grade, and 4.0% in fourth grade.

Like every other cross-sectional database, compared to longitudinal studies, EGD2009 does not allow to obtain causal effects. But, at least, it allows us to speak about associations or effects of the explanatory variables when analysing determinants of grade retention. It does not have information about previous performance of the students or about their health status either. Consequently, we cannot control for these aspects.

### Methods

To analyse which variables increase the probability of being retained in primary education, an econometric model can be used in which the dependent variable is binary, taking value 1 when the student has been retained (in second or fourth grade), and 0 otherwise. (Eq 1) shows the probability of being retained of the i-student at the j-centre, depending on the x_ij_ explanatory variables (see descriptive statistics of all variables at S1 Table).

$$E\left(y_{ij}/x_{ij}\right)=Pr\left(y_{ij}=1/x_{ij}\right)$$ (1)

Using the logit function for turning the expected values of the dependent variable into real numbers the model turns into the following logistic model.

$$logit\left\{Pr\left(y_{ij}=1/x_{ij}\right)\right\}=logit\left\{P_{ij}\right\}=ln\left(\frac{P_{ij}}{1-P_{ij}}\right)=\beta_{0}+\beta_{1}X_{ij}$$ (2)

Hence, the probability of being retained can be expressed like in (Eq 3).

$$Pr\left(y_{ij}=1/x_{ij}\right)=\frac{e^{\beta_{0}+\beta_{1}X_{ij}}}{1+e^{\beta_{0}+\beta_{1}X_{ij}}}$$ (3)

The EGD2009 sample, as explained above, has two levels (students and centres). Thus, the logistic model should be expressed like a multilevel one, so that the complete model shall be a multilevel logistic regression model. Including a centre-specific random intercept *ζ*_*j*_~*N*(0, Ψ) in the linear prediction, to relax the assumption of conditional independence among the responses for the same centre given de covariates [60], its specification is the following (Eq 4).

$$\begin{array}{l} \begin{array}{c} logit\left\{Pr\left(y_{ij}=1/x_{pij},\zeta_{j}\right)\right\}=logit\left\{P_{ij}\right\}\;=\;ln\left(\frac{P_{ij}}{1-P_{ij}}\right)\;=\;\beta_{0j}+\beta_{pj}X_{pij}\\ \beta_{0j}\;=\;\gamma_{00}+\gamma_{0q}Z_{qj}+\zeta_{0j}\\ \beta_{pj}\;=\;\gamma_{p0} \end{array}\\ \to logit\left\{Pr\left(y_{ij}=1/x_{pij},\zeta_{j}\right)\right\}=\gamma_{00}+\gamma_{0q}Z_{qj}+\gamma_{p0}X_{pij}+\zeta_{0j} \end{array}$$ (4)

Where P_ij_ is the probability of being retained in primary education of the i-student at the j-centre. This probability is built by β_0j_ (mean probability at the j-centre) and X_pij_ (p-explanatory variables related to individual and family characteristics). β_0j_, in turn, comprises γ_00_ (mean probability of all centres) and *ζ*_0j_ (deviation of the probability of the j-centre to the mean probability of all centres). Finally, Z_qj_ comprises the q-variables related to the centre level.

The sample lets us differentiate two types of retained students. First, those retained in second grade, and second, those retained in fourth grade. Thus, the endogenous variable can be categorized into three categories: Not retained (k = 0), retained in second grade (k = 1) and retained in fourth grade (k = 2). This differentiation makes it possible to analise the main differences between being retained in second or in fourth grade of primary education. Estimating the following two-level multinomial logistic regression (Eq 5) allows us to identify if there are explanatory variables that influence the probability of being retained in each grade differently:

$$\begin{array}{l} \begin{array}{c} logit\left\{Pr\left(y_{ij}\;=\;k/x_{pij},\zeta_{jk}\right)\right\}\;=\;logit\left\{P_{ijk}\right\}\;=\;ln\left(\frac{P_{kij}}{P_{0ij}}\right)\;=\;\beta_{0jk}+\beta_{pjk}X_{pij}\\ \beta_{0jk}\;=\;\gamma_{00k}+\gamma_{0qk}Z_{qj}+\zeta_{0jk}\\ \beta_{pjk}\;=\;\gamma_{p0k} \end{array}\\ \to logit\left\{Pr\left(y_{ij}=k/x_{pij},\zeta_{jk}\right)\right\}\;=\;\gamma_{00}+\gamma_{0qk}Z_{qj}+\gamma_{p0k}X_{pij}+\zeta_{0jk} \end{array}$$ (5)

Both multilevel random-intercept logistic regressions, Eqs (4) and (5), are estimated by maximum likelihood using weights and adaptive quadrature with the statistical software package Stata 13, by using the command gllamm [60].

The EGD2009 data show missing values for some of the considered variables. However, due to the uncertainty of the quality of the assignment of such values [61], we have decided not to use multiple assignment techniques, even if there is a certain loss of sample size, since the estimations do not vary when excluding the observations with missing values [20].

## Results

### Descriptive Analysis

Grade retention is a matter of concern as it is observed that retained students usually performe worse than their non-retained peers at international evaluations, such us those of PISA [62, 63], TIMMS or PIRLS [50, 64]. The same applies to the fourth grade students of the EGD2009. Whether retained or not, we can observe that, no matter when the retention took place –in second or fourth grade of primary–, it brings worse results in all evaluated competences than their non-repeating peers’ (Table 1).

**Table 1:** Scores by competences and grade retention.

Competence	Retained in 2^nd^ grade	Retained in 4^th^ grade	Not retained
**Communicative linguistic competence (CLC)**	420	443	509
**Mathematic competence (MC)**	425	445	508
**Competence in knowledge and interaction with the physical world (CKIPW)**	426	444	509
**Competence in social skills and citizenship (CSSC)**	426	444	509

**Fig 1:**
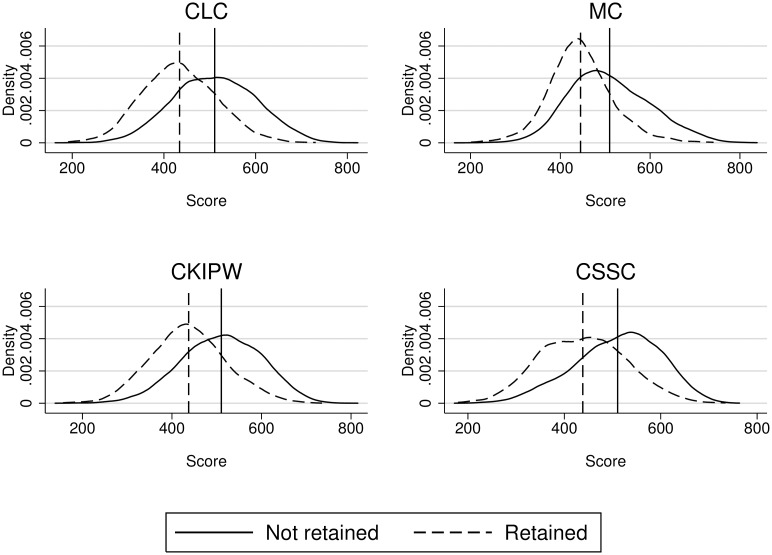
Scores distribution in every competence by retention.

However, the density curves of Fig 1 show that, though the mean score in every competence is higher for the students that were not retained, there are also retained students with better scores than non-retained ones. For that reason there is no clear and direct relationship between retention and performance in the tests, as some authors may suggest, for the scores in the tests cannot be explained just by the condition of being a retained student.

**Fig 2:**
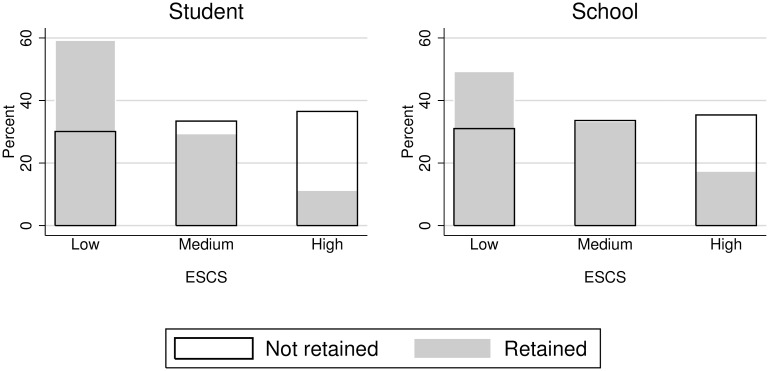
Students’ distribution by socioeconomic and cultural status and grade retention.

Analysing together grade retention and SES (Fig 2), the highest proportion of retained students are clearly those with low socioeconomic and cultural status. Schools with low socioeconomic and cultural status also have the highest proportion of retained students. Among the non-retained students, the distribution is exactly the opposite, but without large differences among the three categories of SES.

**Fig 3:**
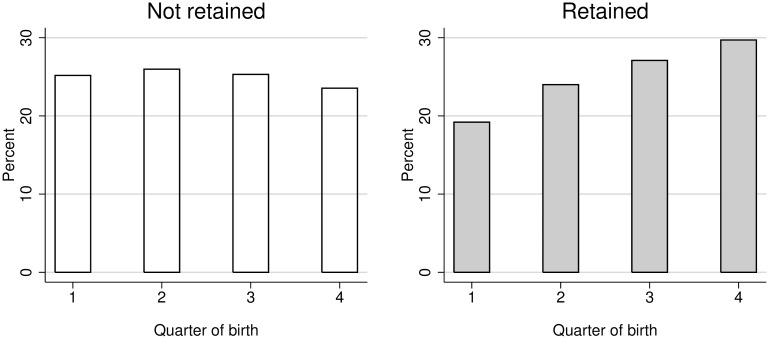
Students’ distribution by retention and quarter of birth.

There also seems to be a clear pattern related to the quarter of birth of the student and having been retained (Fig 3). The distribution of retained students is asymmetric to the left, so that the highest proportion of retained students are the youngest among their cohort (in Spain, those born during the last quarter of the year). However, the distribution of non-retained students is platykurtic, with an equitable distribution among the four quarters of birth.

**Fig 4:**
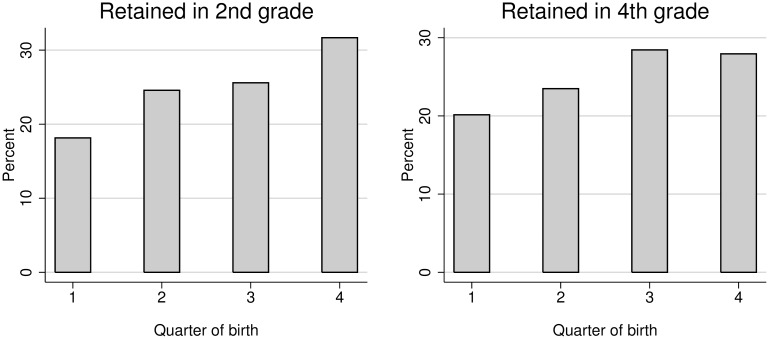
Students’ distribution by grade retention in 2^nd^ and 4^th^ grade and quarter of birth.

Splitting the sample of retained students in two parts, according to the grade in which the retention took place, the same pattern can be observed (Fig 4). There are more retained students among those who were born in the last quarter of the year (the youngest of their cohort), and this is still more pronounced among the students that were retained in second grade.

### Logistic models

The descriptive analysis tries to make an approximation to the common characteristics of retained students, but it is difficult to draw clear conclusions about the variables that really influence the probability of retention without a more complex analysis that includes the *caeteris paribus* condition. The most appropriate econometric model for that aim is the multilevel binomial random-intercept logistic regression (Eq 4). With its estimation it is easy to interpret which variables increase the probability of being a retained student, and which other variables decrease this probability. As most explanatory variables are categorical, Table 2 shows the estimations in the form of odd-ratios. When the odd-ratio is greater (lower) than one, the variable increases (decreases) the probability of grade retention.

**Table 2:** Two-level random-intercept binomial logistic regression.

Level	Variables	O.R.^a^	(95% CI)	p-value
	Constant	0.05	(0.03–0.08)	0.000
Level 1 (Students / Families)				
	Boys (ref. Girls)	1.38	(1.23–1.55)	0.000
	Early schooling (ref. No)	0.86	(0.77–0.97)	0.011
	Quarter of birth… (ref. First)			
	…Second	1.22	(1.03–1.44)	0.020
	…Third	1.45	(1.22–1.72)	0.000
	…Fourth	1.71	(1.45–2.01)	0.000
	SES	0.54	(0.50–0.58)	0.000
	Single-parent family (ref. Nuclear)	1.64	(1.42–1.89)	0.000
	Different language at home and at school (ref. Same)	1.16	(0.98–1.37)	0.076
	Immigrant… (ref. Native)			
	…1st generation	1.97	(1.71–2.28)	0.000
	…2nd generation	1.69	(1.28–2.23)	0.000
Level 2 (Schools / Teachers)				
	Private school (ref. Public)	0.96	(0.79–1.16)	0.669
	Number of students in school	1.00	(1.00–1.00)	0.464
	Teacher training program about…			
	… curricular and methodological issues	0.99	(0.91–1.08)	0.825
	… diversity, coexistence, and interculturality	0.99	(0.91–1.08)	0.849
	… new technologies (ICT)	0.85	(0.74–0.99)	0.037
	Class size (ref. 25 students or less)	1.00	(0.84–1.18)	0.963
	Mean SES of the class… (ref. Low level)			
	…Medium level	0.97	(0.83–1.14)	0.732
	…High level	0.72	(0.57–0.90)	0.004
	Percentage of inmigrant students… (ref. Less than 10%)			
	…Between 10% and 22%	0.93	(0.79–1.10)	0.388
	…More than 22%	0.87	(0.71–1.05)	0.144
	Students’ absenteeism harms learning… (ref. Not at all or very little)			
	…A lot or very much	1.04	(0.90–1.22)	0.588
	Autonomous regions… (ref. Canary Islands)			
	…Andalusia	0.77	(0.51–1.18)	0.239
	…Aragon	1.15	(0.80–1.64)	0.455
	…Asturias	0.74	(0.50–1.10)	0.138
	…Balearic Islands	1.23	(0.87–1.74)	0.237
	…Cantabria	0.84	(0.55–1.30)	0.438
	…Castile—La Mancha	1.17	(0.82–1.68)	0.384
	…Castile and Leon	0.84	(0.56–1.26)	0.400
	…Catalonia	0.33	(0.19–0.55)	0.000
	…Valencia	0.51	(0.34–0.76)	0.001
	…Extremadura	0.68	(0.44–1.06)	0.092
	…Galicia	0.68	(0.45–1.03)	0.066
	…Madrid	1.18	(0.81–1.71)	0.390
	…Murcia	0.78	(0.53–1.15)	0.214
	…Navarre	0.87	(0.59–1.28)	0.490
	…Basque Country	0.97	(0.66–1.41)	0.858
	…La Rioja	0.64	(0.43–0.95)	0.028
	…Ceuta	0.89	(0.54–1.48)	0.662
	…Melilla	0.85	(0.50–1.44)	0.537

Endogenous variable: Probability of being vs. not being retained

^a^ O.R.: Odd ratios

Among the variables related to the students’ level that increase the probability of grade retention, no matter if it took place in second or in fourth grade, the following stand out: being a boy, being born in the second quarter of the year or later, speaking a different language at home and at school, belonging to a single-parent family or being a first or second-generation immigrant. Among the variables related to the school level, no considered variable increases the probability of retention.

On the other hand, the variables of students’ level that decrease the probability of grade retention are: having gone to school before three years old (early schooling) as well as belonging to a family with high SES. At the school level, among all considered variables, there are just two variables that decrease the probability of grade retention: teachers taking part in a training program related to ICT; and the class having a high mean of SES.

We included regional dummies in the model, as there are stark differences across regions and education is highly decentralized in Spain. We left the Canary Islands as reference category, as it is the autonomous region with the highest rate of retention (appart from Ceuta and Melilla) and with one of the lowest results at the EGD2009. Compared to the Canary Islands, students of Catalonia, Valencia, Extremadura, Galicia and La Rioja have less probability of being retained. Other autonomous regions show no statistically significant difference compared to the Canary Islands.

The SES variable is an index at the EGD2009. In (59) it is explained that it was built on a subset of family aspects, such as education level and occupation of the parents, number of books and other educational resources –like having a quiet place to study, Internet access, reading books, and TVs– at home. The first estimated model (Table 2) shows that this index influences the probability of grade retention, but cannot differentiate which exact variables among all components of the socio-economic and cultural background of the student really influence that probability. To address this issue, we estimated again (Eq 4) with the same variables as in Table 2, but instead of using the SES index, we disaggregated it in its own components. Table 3 shows only the odd-ratios for the different components of the SES, though all other variables were also introduced in the model.

**Table 3:** Decomposing the SES (two-level random-intercept binomial logistic regression).

Variables	O.R.^a^	(95% CI)	p-value
Education level of the father… (ref. Less than lower secondary education)			
…Between lower secondary education and post-secondary non-terciary education	0.72	(0.60–0.86)	0.000
…Terciary education	0.43	(0.32–0.59)	0.000
Education level of the mother… (ref. Less than lower secondary education)			
…Between lower secondary education and post-secondary non-terciary education	0.47	(0.39–0.56)	0.000
…Terciary education	0.22	(0.16–0.30)	0.000
Occupation of the father… (ref. Works outside the home)			
…Works from home	1.20	(0.57–2.55)	0.627
…Does not work	1.40	(1.17–1.67)	0.000
Occupation of the mother… (ref. Works outside the home)			
…Works from home	0.94	(0.80–1.11)	0.480
…Does not work	1.24	(1.02–1.51)	0.032
Number of books in the home… (ref. 25 or less)			
…Between 26 and 50	0.80	(0.66–0.97)	0.022
…Between 51 and 100	0.71	(0.58–0.86)	0.000
…Between 101 and 150	0.61	(0.48–0.77)	0.000
…More than 150	0.45	(0.35–0.56)	0.000
Home educational resources	0.92	(0.85–0.98)	0.017

Endogenous variable: Probability of being vs. not being retained. All other covariables of Table 2 were also included

^a^ O.R.: Odd ratios

The higher the education level of both parents and the more books and other educational resources there are in the home, the lower the probability of grade retention. Yet, when either the father or the mother does not work, the probability of grade retention increases.

The students of EGD2009 could have been retained in second or fourth grade of primary. That means that the variable of analysis could have been considered as a categorical one with three different alternatives: not retained, retained in second grade and retained in fourth grade. This leads us to estimate a multinomial logistic regression (Eq 5), instead of a binomial logistic one. Table 4 shows the results of this logistic regression and enables making comparisons of the influence of all covariables on the probability of grade retention differentiating the grade it took place in.

**Table 4:** Two-level random-intercept multinomial logistic regression.

		Retained in 2^nd^ grade			Retained in 4^th^ grade		
Level	Variables	O.R.^a^	(95% CI)	p-value	O.R.^a^	(95% CI)	p-value
	Constant	0.02	(0.01–0.04)	0.000	0.03	(0.02–0.05)	0.000
Level 1 (Students / Families)							
	Boys (ref. Girls)	1.64	(1.39–1.94)	0.000	1.20	(1.03–1.40)	0.021
	Early schooling (ref. No)	0.82	(0.69–0.96)	0.016	0.90	(0.78–1.05)	0.170
	Quarter of birth… (ref. First)						
	…Second	1.28	(1.01–1.63)	0.041	1.17	(0.93–1.45)	0.174
	…Third	1.43	(1.11–1.84)	0.005	1.46	(1.18–1.80)	0.000
	…Fourth	1.93	(1.53–2.43)	0.000	1.53	(1.24–1.89)	0.000
	SES	0.51	(0.46–0.56)	0.000	0.56	(0.51–0.61)	0.000
	Single-parent family (ref. Nuclear)	1.33	(1.08–1.65)	0.007	1.92	(1.61–2.29)	0.000
	Different language at home and at school (ref. Same)	1.14	(0.92–1.42)	0.239	1.18	(0.95–1.48)	0.136
	Immigrant… (ref. Native)						
	…1st generation	1.90	(1.56–2.31)	0.000	2.04	(1.69–2.47)	0.000
	…2nd generation	1.36	(0.91–2.02)	0.132	2.04	(1.42–2.91)	0.000
Level 2 (Schools / Teachers)							
	Private school (ref. Public)	0.85	(0.66–1.10)	0.208	1.05	(0.84–1.32)	0.648
	Number of students in school	1.00	(1.00–1.00)	0.671	1.00	(1.00–1.00)	0.473
	Teacher training program about…						
	… curricular and methodological issues	1.01	(0.91–1.13)	0.801	0.97	(0.88–1.07)	0.530
	… diversity, coexistence, and interculturality	0.99	(0.89–1.09)	0.799	1.00	(0.90–1.11)	0.991
	… new technologies (ICT)	0.88	(0.72–1.06)	0.182	0.84	(0.70–1.00)	0.048
	Class size (ref. 25 students or less)	0.92	(0.74–1.15)	0.457	1.07	(0.88–1.30)	0.502
	Mean SES of the class… (ref. Low level)						
	…Medium level	0.89	(0.71–1.10)	0.281	1.05	(0.87–1.27)	0.595
	…High level	0.70	(0.51–0.97)	0.031	0.73	(0.55–0.95)	0.022
	Percentage of immigrant students… (ref. Less than 10%)						
	…Between 10% and 22%	1.03	(0.82–1.30)	0.774	0.86	(0.71–1.05)	0.143
	…More than 22%	1.03	(0.89–1.34)	0.842	0.75	(0.59–0.95)	0.017
	Students’ absenteeism harms learning… (ref. Not at all or very little)						
	… A lot or very much	1.12	(0.92–1.37)	0.256	0.97	(0.81–1.16)	0.745
	Autonomous regions… (ref. Canary Islands)						
	…Andalusia	0.69	(0.39–1.22)	0.201	0.84	(0.52–1.36)	0.480
	…Aragon	1.03	(0.62–1.69)	0.921	1.24	(0.81–1.89)	0.322
	…Asturias	0.92	(0.55–1.56)	0.766	0.60	(0.37–0.97)	0.038
	…Balearic Islands	1.63	(1.02–2.59)	0.041	0.91	(0.60–1.39)	0.671
	…Cantabria	0.66	(0.38–1.14)	0.138	0.98	(0.59–1.63)	0.942
	…Castile—La Mancha	0.94	(0.57–1.53)	0.790	1.37	(0.92–2.06)	0.124
	…Castile and Leon	0.83	(0.48–1.44)	0.499	0.85	(0.50–1.47)	0.568
	…Catalonia	0.45	(0.25–0.83)	0.011	0.23	(0.11–0.46)	0.000
	…Valencia	0.42	(0.24–0.76)	0.004	0.58	(0.37–0.91)	0.018
	…Extremadura	0.74	(0.41–1.35)	0.326	0.63	(0.34–1.15)	0.135
	…Galicia	0.49	(0.29–0.84)	0.010	0.85	(0.52–1.37)	0.498
	…Madrid	1.08	(0.64–1.80)	0.781	1.26	(0.80–1.99)	0.310
	…Murcia	0.74	(0.43–1.25)	0.258	0.81	(0.51–1.29)	0.379
	…Navarre	0.97	(0.58–1.62)	0.914	0.79	(0.50–1.25)	0.316
	…Basque Country	1.33	(0.82–2.18)	0.247	0.69	(0.40–1.18)	0.171
	…La Rioja	0.73	(0.44–1.22)	0.230	0.56	(0.35–0.91)	0.018
	…Ceuta	1.02	(0.57–1.83)	0.952	0.77	(0.45–1.33)	0.346
	…Melilla	0.92	(0.46–1.87)	0.824	0.77	(0.45–1.34)	0.361

Endogenous variable: Probability of grade retention in 2^nd^ (4^th^) grade vs. not being retained

^a^ O.R.: Odd ratios

There are some variables with nearly the same influence in the probability of grade retention in both groups –those retained in second and those retained in fourth grade– like the students’ SES, being a first-generation inmigrant and having a high mean SES in the class, but others differ among groups.

There are variables whose influence is higher in the probability of grade retention in second grade than in fourth grade, such as being a boy or being born in the fourth quarter of the year. On the other hand, belonging to a single-parent family increases the probability of grade retention to a greater extent in the fourth than in second grade.

There are also variables that influence the probability of grade retention in second grade, but do not influence the probability of grade retention in fourth grade any more, like being born in the second quarter of the year or having gone to school before three years old.

Finally, there are variables that influence only the probability of grade retention only in fourth grade, like being a second-generation immigrant, going to a school with teacher training programs about new technologies or the percentage of immigrant students in the class. The estimations show that the higher proportion of immigrants in the class, the less probability of being retained, maybe because of the lower expectations of the teachers for the whole class.

Among the variables related to the student level we see that early schooling reduces the probability of being retained in second grade, but it is not statistically significant for explaining the probability of grade retention in fourth grade. Though it does influence the results in both grades [11], it does not influence grade retention in fourth grade any more. Maybe, as the literature says [28, 29, 65], most of the children that were retained in second grade could have had just learning difficulties, and the variable ‘early schooling’ could be mitigating this effect, enhancing their results and lowering the probability of failure and consequently the probability of grade retention.

The regional dummies included in the model also show different behaviours related to second and fourth grade retention, compared to students living in the Canary Islands. Thus students living in the Balearic Islands (Galicia) have a higher (lower) probability of being retained in second grade. Speaking about fourth grade retention, students living in Asturias or La Rioja, show less probability of grade retention in fourth grade. Finally, students living in Catalonia or Valencia have less probability of grade retention in both grades.

Though the students’ SES similarly influences the probability of grade retention in both groups, there may be differences in the influence of its components in each group. In order to check this, we estimated again (Eq 5) with the same variables as in Table 4, but disaggregating the SES index in its own components. Table 5 shows only the odd-ratios for these components, though all other variables were also introduced in the model.

**Table 5:** Decomposing the SES (two-level random-intercept multinomial logistic regression).

	Retained in 2^nd^ grade			Retained in 4^th^ grade		
Variables	O.R.^a^	(95% CI)	p-value	O.R.^a^	(95% CI)	p-value
Education level of the father… (ref. Less than lower secondary education)						
…Between lower secondary education and post-secondary non-terciary education	0.81	(0.62–1.04)	0.101	0.64	(0.51–0.81)	0.000
…Terciary education	0.45	(0.29–0.70)	0.000	0.42	(0.28–0.62)	0.000
Education level of the mother… (ref. Less than lower secondary education)						
…Between lower secondary education and post-secondary non-terciary education	0.37	(0.28–0.48)	0.000	0.60	(0.47–0.76)	0.000
…Terciary education	0.20	(0.13–0.30)	0.000	0.24	(0.16–0.37)	0.000
Occupation of the father… (ref. Works outside the home)						
…Works from home	0.89	(0.27–2.95)	0.844	1.55	(0.63–3.82)	0.346
…Does not work	1.48	(1.14–1.91)	0.003	1.33	(1.04–1.70)	0.023
Occupation of the mother… (ref. Works outside the home)						
…Works from home	1.01	(0.80–1.27)	0.954	0.89	(0.71–1.11)	0.287
…Does not work	1.18	(0.88–1.57)	0.272	1.30	(1.01–1.67)	0.039
Number of books in the home… (ref. 25 or less)						
…Between 26 and 50	0.86	(0.67–1.12)	0.257	0.75	(0.58–0.96)	0.025
…Between 51 and 100	0.72	(0.54–0.95)	0.022	0.69	(0.52–0.90)	0.007
…Between 101 and 150	0.58	(0.40–0.82)	0.003	0.62	(0.46–0.85)	0.003
…More than 150	0.50	(0.36–0.69)	0.000	0.40	(0.29–0.5)	0.000
Home educational resources	0.91	(0.82–1.01)	0.076	0.92	(0.84–1.01)	0.090

Endogenous variable: Probability of grade retention in 2^nd^ (4^th^) grade vs. not being retained. All other covariables of Table 4 were also included

^a^ O.R.: Odd ratios

As expected, there are some differences related to the influence of the SES components in the probability of grade retention in second and fourth grade. The influence of the father’s education level is higher in the probability of grade retention in fourth grade than in second grade. On the contrary, the influence of the mothers’ education level is higher in the probability of retention in second grade. Related to occupation, it is just the reverse. Having an unemployed father increases the probability of second grade retention, and having an unemployed mother only influences the probability of fourth grade retention. Finally, related to the number of books and other educational resources at home, there are no real differences between both groups of students.

## Discussion and Conclusion

The main objective of this paper was to detect which variables –related to students and schools– could increase or decrease the probability of ‘early’ grade retention, that is, retention in primary education. Moreover, we also wanted to examine whether these variables equally influenced the probability of being retained in second and in fourth grade. Another objective was to analyse in detail the effect of socioeconomic status on the probability of early grade retention, to be able to draft which specific aspects of the SES are more likely to influence it.

We have focused on early grade retention for two reasons. First, because there is a lot of comprehensive and valuable information at the EGD2009. In it, unlike in other similar evaluations like PISA, PIRLS and TIMSS, repeaters are clearly identified and, besides, one can distinguish between those who had repeated second grade of those who were repeating fourth grade –when this evaluation took place–. And second, because grade retention is a practice whose effectiveness has been widely discussed –from a cost-benefit point of view–, in terms of its educational effectiveness as well as to its financial costs.

The used national dataset, with more observations than any other international ones, also allows us to analyse the effect of every Spanish autonomous region on grade retention. Analysing the EGD2009 information we showed that in Spain both group of students (those retained in second and in fourth grade) obtain worse results in all evaluated competences than their non-retained peers. Furthermore, the students that had been retained in second grade obtained worse results than the peers that were repeating fourth grade at the time of the evaluation. Thus, though having been retained so early –in second grade– these students do not improve their school performance two years after their retention, as they still perform worse than all their peers, and therefore the strategy is not achieving the expected results. And the same happens with the students that were repeating fourth grade, as they also perform worse than their peers. All this could have several explanations. First, it is possible that grade retention, as a strategy to give more time to the students to achieve academic objectives, is just a non-effective practice. Second, maybe the question is the way that grade retention is taking place, since it very often means just doing the same things once again, instead of doing them in a different way. Finally, this type of grade retention –so early– could be associated to learning difficulties or even to health problems that had not been diagnosed at that moment.

There is an overlap between the variables that influence academic results according to the EGD2009 [11], and the variables that increase/decrease the probability of grade retention. Thus, boys tend to have a higher probability of grade retention, as well as students that were born after the first quarter of the year, those who belong to single-parent households, and the ones who are the first- or second-generation immigrants. On the contrary, the probability of grade retention dicreases for students who have been ‘early schooled’–before age three–, as well as when the SES increases.

Al the school level there are less variables that influence the probability of grade retention. It decreases only if the teachers take part in a training program related to new technologies, or if the mean SES of the class is high. Finally, the autonomous regions that generally show a lower probability of grade retention, compared to the Canary Islands, are Catalonia and Valencia.

A major finding of this study is the difference detected between second and fourth grade retention in relation to the quarter of birth. In both cases, as the student has been born later in the year of their age cohort, he/she increases his/her grade retention probability. However, it is not a same rate increase. The probability of grade retention in second grade for those born in the fourth quarter of the year –compared to their older peers born in the first quarter– nearly doubles the probability of grade retention in fourth grade. Besides, in fourth grade there is no statistically significant difference between those born in the first and in the second quarter of the year. These results suggest that a part of the total second grade retention can be related to the relative age of the student in their age cohort, and not so much to the students’ competence level or their own capacities.

Deepening in the different variables included in the SES we also detected differences in its influences in the probability of grade retention in both grades. Related to the parents’ level of education, our results show that the mothers’ education level influences more than the fathers’ one –decreasing the probability of grade retention the more education level the mother has– especially in second grade. The influence of the employment status of the parents is quite the reverse. Having an unemployed father increases the probability of grade retention much more than having an unemployed mother, to the extent that there is no statistically significant influence of having an unemployed mother in the probability of grade retention in second grade. Finally, we also observed that the more books there are in the home, the less probability of grade retention, and this influence is higher than the one related to other educational resources at home.

In the light of the results obtained, we make the following three proposals, whose intention is to reduce the early grade retention that is taking place in second and fourth grade of primary education. First, teachers should take the ‘quarter of birth effect’ into consideration, especially among second grade students. That means that it is necessary to respect the maturative development process of the students, and to adapt the expected objectives to this process. Second, it is important to diagnose the possible learning difficulties as early as possible (at least during the first or second grade), as they may be avoiding the positive effect of grade retention in the future academic results. And, third, in relation with the socioeconomic status of the student it is important to bear in mind that it is necessary to develop measures –by the school and the education administration– that try to compensate the effect of belonging to a family whose parents –and especially mothers– have a low academic level, as well as to families with unemployment situations –especially fathers–.

By implementing these three proposals grade retention will tend to decline, mainly retention in second grade. Thus, the future educational achievement of students will improve, and the associated economic savings may be used for other necessary educational measures.

## Supporting Information

S1 Table (DOCX)
