# Supplementary material for: Grade Retention in Primary Education Is Associated with Quarter of Birth and Socioeconomic Status
Source: PLoS One. 2016 Nov 16;11(11):e0166431. doi: 10.1371/journal.pone.0166431 (PMC5112784; doi:10.1371/journal.pone.0166431)
Supplement: S1 Table — (DOCX) [file pone.0166431.s001.docx]

# Supporting information

**S1 Table. Descriptive statistics of the explanatory variables used in the different models**

| **Variables** | **Categories** | **N ^a^** | **Not retained** | **Retained** |
| --- | --- | --- | --- | --- |
| Gender | Girl | 13158 | 93.6 | 6.4 |
|  | Boy | 13622 | 91.1 | 8.9 |
| Early schooling | Before 3 years old | 14884 | 93.7 | 6.3 |
|  | At age 3 or more | 11834 | 90.8 | 9.2 |
| Quarter of birth | First | 6495 | 94.4 | 5.6 |
|  | Second | 6780 | 93.3 | 6.7 |
|  | Third | 6675 | 92.2 | 7.8 |
|  | Fourth | 6295 | 91.0 | 9.0 |
| Socioeconomic status (ESCS) | Low | 8761 | 86.1 | 13.9 |
|  | Medium | 8980 | 93.0 | 7.0 |
|  | High | 9381 | 97.5 | 2.5 |
| Type of family | Nuclear | 23350 | 93.0 | 7.0 |
|  | Single-parent family | 3285 | 87.9 | 12.1 |
| Language at home and at school | The same | 22526 | 93.2 | 6.8 |
|  | Different | 3280 | 90.1 | 9.9 |
| Immigrant | Native | 21769 | 93.9 | 6.1 |
|  | 1^st^ generation | 4641 | 85.4 | 14.6 |
|  | 2^nd^ generation | 712 | 85.8 | 14.2 |
| Type of school | Public | 17380 | 90.8 | 9.2 |
|  | Private | 9742 | 94.9 | 5.1 |
| Number of students in school | 415 students or less | 9046 | 90.7 | 9.3 |
|  | Between 416 and 660 | 8931 | 92.2 | 7.8 |
|  | More than 660 students | 9145 | 93.9 | 6.1 |
| Teacher training programs: curricular issues | No | 9629 | 91.7 | 8.3 |
|  | Yes | 17136 | 92.7 | 7.3 |
| Teacher training programs: methodological issues | No | 12361 | 91.5 | 8.5 |
|  | Yes | 14404 | 93.1 | 6.9 |
| Teacher training programs: diversity | No | 14915 | 92.2 | 7.8 |
|  | Yes | 11850 | 92.5 | 7.5 |
| Teacher training programs: coexistence | No | 14103 | 91.8 | 8.2 |
|  | Yes | 12662 | 93.0 | 7.0 |
| Teacher training programs: interculturality | No | 21698 | 92.4 | 7.6 |
|  | Yes | 5067 | 92.3 | 7.7 |
| Teacher training programs: ICT | No | 11360 | 90.9 | 9.1 |
|  | Yes | 15405 | 93.5 | 6.5 |
| Class size | 25 students of less | 18293 | 91.8 | 8.2 |
|  | More than 25 students | 8359 | 93.4 | 6.6 |
| Mean ESCS of the class | Low | 8798 | 88.1 | 11.9 |
|  | Medium | 9113 | 92.5 | 7.5 |
|  | High | 9211 | 96.2 | 3.8 |
| Percentage of immigrant students | Less than 10% | 8889 | 94.3 | 5.6 |
|  | Between 10% and 22% | 9096 | 92.9 | 7.1 |
|  | More than 22% | 8667 | 89.6 | 10.4 |
| Students’ absenteeism harms learning | Not at all or very little | 19133 | 93.3 | 6.7 |
|  | A lot or very much | 7563 | 89.9 | 10.1 |
| Autonomous regions | Andalusia | 1556 | 91.7 | 8.3 |
|  | Aragon | 1545 | 92.6 | 7.4 |
|  | Asturias | 1469 | 94.0 | 6.0 |
|  | Balearic Islands | 1549 | 89.1 | 10.9 |
|  | Canary Islands | 1576 | 88.6 | 11.4 |
|  | Cantabria | 1484 | 94.1 | 5.9 |
|  | Castile - La Mancha | 1467 | 89.4 | 10.6 |
|  | Castile and Leon | 1315 | 94.1 | 5.9 |
|  | Catalonia | 1472 | 96.5 | 3.5 |
|  | Valencia | 1528 | 95.0 | 5.0 |
|  | Extremadura | 1375 | 92.4 | 7.6 |
|  | Galicia | 1407 | 93.5 | 6.5 |
|  | Madrid | 1622 | 92.5 | 7.5 |
|  | Murcia | 1552 | 91.7 | 8.3 |
|  | Navarre | 1459 | 93.3 | 6.7 |
|  | Basque Country | 1471 | 93.8 | 6.2 |
|  | La Rioja | 1628 | 93.9 | 6.1 |
|  | Ceuta | 881 | 87.8 | 12.2 |
|  | Melilla | 766 | 88.3 | 11.7 |

^a^ N: Number of observations for each variable, without missing values.
